# A Plea for the Preservation of the Natural Teeth

**Published:** 1892-09

**Authors:** W. Pearson

**Affiliations:** Toronto, Ont.


ARTICLE III.

### A PLEA FOR THE PRESERVATION OF THE NATURAL TEETH.

BY W. PEARSON, L. D. S., TORONTO, ONT.

Read before the Ontario Dental Society, Toronto, July, '92,

The Committee on Programme have assigned the above subject to me for a paper. Had they asked me to choose a subject for myself, this certainly would not have been the title. It never would have occurred to me that there existed a disposition to remove natural teeth, to the extent that any special pleading in their behalf would be in good taste or called for. That they have done so leads me to think that there is a necessity for something to be said on the subject. Where it exists, why it exists, and to what extent, I am at a loss to determine. There may be a few, one or two, perhaps a score of dental surgeons in Ontario who need reconstructing. That there are many I do not believe. In all learned professions there will be found some, whose interpretation of the meaning of a word or the sense of a phrase is very different from the generally accepted idea. So we may have those among us who do not exactly grasp the meaning of the word dentistry, more particularly the symbolic combinations of L. D. S., D. D. S., or M. D. S.

That there should at the present day appear to be a necessity, that there should exist an idea of the necessity for a plea for the preservation of natural teeth at this stage of professional progress, is to abandon the whole status and retrograde forty years or more, to disband the profession, as it were, to throw the fat into the fire and call ourselves failures, and we ought to seek some other honest calling in which spheres of usefulness may open up to us, in pursuit of which a just appreciation of our endeavors may be looked for. Is not the sum and substance, the life and vigor, the brains, the whole physical being of the profession, a concentration and embodiment of the principle of the preservation of the natural teeth? To say anything else is a giving away of the fundamental principles.

I apprehend that thirty years ago there did exist a state which, viewed by the light of the present scholastic training, was a deplorable era, marked by the blood of thousands of slaughtered innocents, and might be termed the age of slaughter: in which time the profession was made up of broken-down tradesmen and mechanics, bounty jumpers and refugees from foreign parts, farmers and furriers, plumbers and tinkers, who, for a monetary consideration and six months' service, were turned loose on the community, and permitted to pursue their course according to the light that was in them, and everything was good grist for their mill. Happily, law and order has prevailed over this state of things, and the profession and people are protected.

If I were to say that the practices that then prevailed do now exist even in remote districts to any great extent, would be a-begging the question. I do not believe so. Yet I am led to believe by the action of the committee in the choice of this subject, that there does at the present day and generation, exist somewhere in Ontario one or more of those fossils, or perhaps pupils of the extinct race, who believe that their mission is to mutilate humanity from mercenary motives. I do not expect to teach them any better, that would be too much to expect of them, for as a rule they do not attend conventions since they know it all now. It is far easier on their conscience to stay at home and feel right than to learn better and not be able to do better. The only hope we have of them is that they will soon die and make room for civilized and enlightened beings to take their places.

Where do these people exist? Do we find them in country places, or in the towns and cities? or why do they exist? There must be some demand for them, or do they create the demand? I hold the idea that every graduate in dentistry is by virtue of his qualification an educator. To the extent of his interpretation of the technical teachings of his college days, he must be responsible for his acts and manipulations, which must be reflected in time by the community either to his credit or damage.

If by a careful consideration of a certain case a satisfactory conclusion is arrived at, and a monument of skill and durability is the result, he has commenced to educate the community to his advantage. So according to his leading may he expect to find his patrons following, and if we find an isolated community given to false teeth, the chances are that the dentist is a rubber worker. In large communities we find all sorts and conditions of humanity, the rich and the poor, the educated and the ignorant, those from the rural districts and the city-bred; these call for different classes of the profession to deal with them. They differ in tastes and inclinations. One has the idea that false teeth are the perfection of life, another has a longing for lots of gold to show. Many let their teeth decay because it is cheaper to do so, and so on all through. There is a difficulty here in discriminating conscientiously, and yet there ought not to be. In rural districts, perhaps to a greater extent than in cities, the dentist may reflect his ideas more upon his patrons, for if he is the only representative he may be firm and decisive, argumentative and convincing in just the degree his inclination leads him. If he is an artist in rubber, false teeth is his line of argument, and I am afraid that frequently this result is from inability or indifference, and sometimes, perhaps, from the financial standpoint of the patient, and not from a correct measure of the state of the teeth. I apprehend that there is a tendency all over the country for improvement on the old method of sacrificing natural teeth, both on account of the effect of the superior education at present obtainable at our colleges, and the higher attainments in matriculants, and the good taste and ability to indulge it by the prosperous farmers and artisans, making up the various communities, and aside from individual cases which prove very little, there is a decided change for the better in the way of saving natural teeth.

It does not come within the province of this paper to discuss ways and means of saving teeth, or of the advisability of doing so under exceptional circumstances. These points are left to the superior judgment and intelligence of the conscientious operator to decide. What the writer expects to do is to introduce the subject from a personal standpoint, and invite discussion and criticism, and thus lead up to a consideration of the many points involved. The subject is so wide, so big, so important, that a whole week could be spent on it, and the whole range of the curriculum gone over from Genesis to Revelation, in consideration of means to be adopted and ways to be pursued in saving teeth.

Let me ask you a question here, and let each one of you be prepared to give me an answer of some sort. I know each one of you will have an answer, and each one perhaps a different one, according to his personal practice, subject to local and personal qualification. The question is, When am I justified in using the forceps? Is it in the case of the temporary teeth? No, decidedly not; not under any circumstances, until the age of the child indicates that the time for action has arrived, and which to my mind is not until the new tooth is ready to take the place of the old one. More harm may be done at this age by premature removal than by delaying too long. Judicious treatment and filling (if possible) is always to be resorted to. Have a mind of your own and a policy to pursue and carry it out, and take the responsibility on your own shoulders without regard to parental or childish whims. In removing these teeth I have fallen into the habit of operating chiefly with my fingers or by an incidental application of a probe or excavator, and very seldom indeed resort to forceps, rather preferring to wait until such times as they are not a necessity, and prefer leaving roots until the full time for the new tooth to appear in a few days after operating. Is it in the case of the sixth year molars that I am to begin my maltreating practices, to do evil that good may come of it? This is my opinion in a general way of removing sixth year molars. To make a quotation for the occasion: "To be or not to be, that is the question. Whether it is better to suffer the stings and pains of outraged nature and present troubles, or to take up arms and fly to the evils that we know not of." No dentist is able to determine what is going to be the result of premature extraction of a sixth year molar upon the undeveloped maxillary. The facial derangement is more than he can forsee.

After years of careful observation and study of many cases in regulating by myself and others, where these teeth have been sacrificed and where not, I am strongly convinced that there is an injudicious and wholly unnecessary sacrifice of good teeth here. I shall have to admit that once in a while a case is presented where extraction is advisable, but this is the exception, while too many make it the rule.

It is the shortest way out of a difficulty, the easiest way to settle the question. No account of the future years of lost usefulness, no consideration of facial expression, of the possibility of a contraction of the maxillary, or of a deviation from the plane of the grinding surface of the future arrivals enters into the consideration. It is simply expedient to extract and that ends it for the time being. No ghosts of the slaughtered innocents are likely to arise to trouble the conscience or rob us of innocent repose. Notwithstanding all this the principle is wrong. Conceived in ignorance and born in iniquity, it is practised too much and ought to be discontinued. Nature never provided a more fitting object for man's good, at a more opportune time, in a better place, than this same tooth, and am I, the learned and intelligent fellow-being who, by choice in a scientific specialty, is referred to by reason of my standing and experience, justified when I say I can do nothing but extract? I think not. Or does it justify me when I say, "Oh, yes, I might perhaps do something for you. I might save the tooth for a few years, but ultimately you may lose it. You had better lose it now and later on you won't miss it much." This looks like prostitution to me, and I prefer to save the semblance of a sixth year molar, at all events for a few years, until nature provides another to take its place to carry on the great work for which they are so vitally essential, which you all very well understand, and so much longer as skill and modern advanced dentistry may enable me to.

Use your skill and resources on these teeth without regard to remuneration or desire of the patient. It does not excuse you to say that it is ulcerated or the nerve is dead, or that the patient is poor, or ignorant, or unappreciative. Save the tooth and put it down to charity, which may cover a multitude of sins otherwise laid up against you. As far as individual cases of extracting are concerned, as they are presented to the dentist for relief from present pain, and where the denture is not the immediate question. I apprehend that there is no difference of opinion, that all modern operators do make an attempt, and generally successfully, to save such a case. The point of debate and hesitation is generally when a few of the teeth are in need of treatment, or in case of a few good ones remaining, and the others more or less involved in doubt as to the advisability of attempting their salvation. In the light of present progressive dentistry, we can scarcely be excused in our action if we recommend a resort to extraction, except in cases of badly wasted roots. I hold a strong prejudice against removing even sound roots, preferring to fill them where they cannot be crowned, and protecting the soft tissue and upholding the alveolus as long as possible. A sound root may be serviceable for years, especially so after treatment and filling or capping.

Looking at the æsthetic effects of removing teeth and restoring by the factory-made article, I presume that many will consider me wild when I make the assertion, that it is a physical impossibility to restore or reproduce the natural expression to a face when once the roots of the teeth are removed. Yet, I make the statement, and challenge the artist in dentistry to get up and say so. It cannot be done.

The canine eminence cannot be prolonged on the outside of the maxillary sufficiently high, without interfering with the free action and motion of the lips. As soon as the six anterior teeth are removed, there begins a change in the body of the jaw as well as the alveoli, too high up for any outside artificial contrivances to be placed for the comfort of the patient.

It may be possible that this is the reason why our English brethren do not, as a rule, remove the roots in substituting the natural teeth; and, if so, I commend them for their good taste, from an artistic point of view, while from a sanitary or economic point, perhaps there is not so much to be said in its favor.

My faith in the dentist of the present and of the near future is unbounded, as to their action in regard to saving teeth. Everything is promising, their inclination is in that direction, their education is directed in that way, public taste is being directed more in that way, humanity calls upon them to do so, progressive ideas must prevail, and the time is coming when the forceps will be a quarterly or semi-annual issue, instead of a daily. This will be brought about by honest, intelligent application of ways and means of treating and preserving the teeth. Honest endeavor and individual enterprise will help the public to see the folly of making needless sacrifice. Honest dentists will help to make honest and intelligent patrons.

Intelligent patrons will demand the best of service, and if honest to themselves and the profession, they will not be rummaging the newspapers to find the Cheap Johns who advertise eight dollar teeth (and gas free until the first of the month). Unfortunately for both the public and the profession, the curse of dentistry of the present day is this demoralizing system of advertising cheap work.

If I were to be told that a party had administered, on an average, gas six times a day, for a whole twelve months or two years, I should unhesitatingly say that that party was a conscienceless humbug, and was not practising dentistry at all, but prostituting a noble calling for filthy lucre. No doubt would exist in my mind that hundreds of good teeth must have been removed to be substituted by china store teeth.

This difficulty is difficult to regulate and can only be done by a firm determination upon the part of each and every new graduate to live up to the standard of professional etiquette, and adhere to the moral code of ethics, so generally understood if not always expressed.

A personal sense of honor and right pervades every properly constituted gentleman, and while he obeys this better instinct there is not much need for a written code to be placed before him.

There is much need in this country, in all countries, for advanced, scientific, conscientious dentists, who are willing to climb the hill of fame step by step, and by carefully laying the foundation of success upon correct principles, build up a reputation which will be lasting and enviable, but it can never be done on the line of false teeth, nor by printer's ink. If you know of any young man, graduate or undergraduate, bursting with ambition to do it all, given to big head, capable of running the whole concern, without much moral restraint and no conscience, utterly selfish, for whom the world was made and without whom the earth would be a desolation, you might advise him to put money into ink slinging and cheap teeth for his theme; but to any of my hearers who regard honor above any other external advantage, who have any regard for the opinion of good and true men, and who would care to establish a reputation on substantial grounds, avoid the stumbling-block of cheap teeth and advertising. The success attending this line of procedure is of the most ephemeral nature, requiring a constant resort to the same artificial stimulant, while without doubt it ostracises the operator from association and intercourse with such as might be a benefit, and it does not catch those of the public whose patronage and influence are the mainstay of a lucrative practice. It appeals to the most degrading sense of humanity, and leads the ignorant, thoughtless victim to expect good results without adequate remuneration, a principle which the commonest laborer, from the hod-carrier to the 'longshoreman and washee washee Chinaman, has long since recognized as vicious and wrong. We would perhaps not be interested in this disgraceful, unprofessional, unsound and undignified way of boasting along an unhealthy and undesirable practice, if it were not for the pernicious and totally uncalled-for sacrifice of good teeth which it involves, and the false impressions and wrong views it fastens upon the poor victims, who are led to measure the standard of the profession by their experience with these snappers and whippers-in whose failures and misdeeds are laid up to the general account too frequently.

It may be possible that this idea of demoralizing degeneracy is an outcome of too much competition, of too much supply for the demand for professionals, and the resulting sickly and unsatisfactory growth of practice, but my own opinion is not that such is the case. Since we will always find room at the top for good men, and the demand is always for more of such, and welcome to them with both hands held up.

What the public and professional demand of the day is, for less indiscriminate slaughter of good teeth, and more conservative treatment and preservation along the lines of advanced ideas and present day possibilities.—*Dominion Dent. Journal.*

---

ARTICLE IV.

### CONSTRUCTING CROWN-AND-BRIDGE-WORK.

